# Association of *TGF-β1* and *IL-10* Gene Polymorphisms with Osteoporosis in a Study of Taiwanese Osteoporotic Patients

**DOI:** 10.3390/genes12060930

**Published:** 2021-06-18

**Authors:** Min-Yu Tu, Kuei-Yang Han, Ying-Wei Lan, Ku-Yi Chang, Cheng-Wei Lai, Theresa Staniczek, Chung-Yu Lai, Kowit-Yu Chong, Chuan-Mu Chen

**Affiliations:** 1 Department of Life Sciences and Ph.D. Program in Translational Medicine, National Chung Hsing University, Taichung 402, Taiwan; du0807@yahoo.com.tw (M.-Y.T.); bublelanwilliam@gmail.com (Y.-W.L.); parenchyem@yahoo.com.tw (C.-W.L.); Theresa.Staniczek@medma.uni-heidelberg.de (T.S.); 2 Department of Health Business Administration, Meiho University, Pingtung 912, Taiwan; 3 Institute of Biomedical Science and Technology, National Sun Yat-Sen University, Kaohsiung 804, Taiwan; 4 Kaohsiung Armed Forces General Hospital Gangshan Branch, Kaohsiung 820, Taiwan; 5 Department of Family Medicine, Jen-Ai Hospital, Dali Branch, Taichung 402, Taiwan; shinchen@livemail.tw; 6 Department of Orthopedic Surgery, Jen-Ai Hospital, Dali Branch, Taichung 402, Taiwan; smallhead1230@yahoo.com.tw; 7 Department of Dermatology, Venereology and Allergology, University Medical Center and Medical Faculty Mannheim, and Center of Excellence in Dermatology, Heidelberg University, Mannheim 69117, Germany; 8 Graduate Institute of Aerospace and Undersea Medicine, National Defense Medical Center, Taipei 114, Taiwan; multi0912@gmail.com; 9 Department of Medical Biotechnology and Laboratory Science and Graduate Institute of Biomedical Sciences, Division of Biotechnology, College of Medicine, Chang Gung University, Taoyuan 333, Taiwan; 10 Hyperbaric Oxygen Medical Research Lab, Bone and Joint Research Center, Department of Laboratory Medicine, Chang Gung Memorial Hospital, Linkou, Taoyuan 333, Taiwan; 11 The iEGG and Animal Biotechnology Center and the Rong Hsing Research Center for Translational Medicine, National Chung Hsing University, Taichung 402, Taiwan

**Keywords:** osteoporosis, single-nucleotide polymorphism (SNP), anti-inflammatory cytokine, transforming growth factor-β1 (TGF-β1), interleukin-10 (IL-10), bone mineral density (BMD)

## Abstract

Osteoporosis is a rising health threat in the increasingly aging world population. It is a common skeletal disease strongly linked to genetic predisposition. We aim to identify the effects of the anti-inflammatory *TGF-β1*- and *IL-10*-specific single-nucleotide polymorphism (SNP) combination on the risk for osteoporosis. We investigated and analyzed the relationships between three *TGF-β1* SNPs (−509C/T, +869 T/C and +29T/C), one *IL-10* SNP (+1927A/C) and the level of bone mineral density (BMD), as well as the risk of osteoporosis in Taiwanese osteoporotic patients. A total of 217 subjects were recruited, including 88 osteoporotic patients and 129 healthy controls, for SNPs, BMD and clinical characteristics statistical analyses. Females with *TGF-β1* SNP (−509 C/C) and *IL-10* SNP (+1927 C/C) genotypes showed a great benefit for femoral neck T-scores. However, the combination of *TGF-β1* SNP (−509 T/T) and *IL-10* SNP (+1927 A/A) genotypes in all subjects showed a significant decrease in total hip BMD T-scores. The *TGF-β1* SNP (−509 C/T) genotype in all subjects and *TGF-β1* SNP (−509 T/T) and *IL-10* SNP (+1927 A/C) genotypes in males showed positive effects on body height. The combination of the many SNPs in the anti-inflammatory *TGF-β1* and *IL-10* genes may be cooperatively involved in the development of osteoporosis. Our data suggested that the specific SNP combination of *TGF-β1* (−509) and *IL-10* (+1927) may act as a predictive factor for postmenopausal osteoporosis in Taiwanese women.

## 1. Introduction

Osteoporosis is a common bone disease in humans and is rapidly increasing to become a major public health problem among elderly individuals worldwide [1]. Osteoporosis is defined as low bone mineral density and the disruption of the bone microarchitecture, with a consequent increase in bone fragility and decrease in bone strength, increasing the risk of fracture [2]. Bone strength is strongly associated with the interaction between different regulatory factors, including genetic, epigenetic, environmental and behavioral factors [3]. Several twin and family studies also support the finding that over 50% of the variance in bone mass and fracture risk is genetically determined [4,5,6].

Several candidate genes and their SNPs are strongly associated with osteoporosis pathogenesis, including transforming growth factor-β1 (*TGF-β1*), interleukin-10 (*IL-10*), vitamin D receptor (*VDR*), osteoprotegerin (*OPG*), collagen type I α 1 (*COL1A1*) and estrogen receptor (*ER*) genes, and may act as potential clinical markers for osteoporosis [7,8,9].

*TGF-β1* is known as a strong candidate gene for the study of osteoporosis, because of the modulation and maintenance of TGF-β1 in balancing between bone resorption and the bone formation process [10]. TGF-β1 is synthesized by osteoblasts and is abundant in bone, where it can stimulate preosteoblast proliferation or differentiation, block osteoblasts apoptosis, and act as a chemoattractant to recruit osteoblastic precursors or osteoblasts. On the other hand, TGF-β1 had a biphasic effect on osteoclast maturation depending on its concentration; i.e., low concentrations of TGF-β1 promoted osteoclast maturation and vice versa [11,12,13,14]. Geiser et al. [15] observed a decrease in tibia length and a reduction in bone density in *tgf-β1* knockout mice. Several polymorphisms have been described in the coding, introns or intergenic sequences of the *TGF-β1* gene and some of them have been associated with functional implications on TGF-β1 production and disease susceptibility [16,17]. In a study of twins, Grainger et al. [18] found that approximately 8.2% of the additive genetic variance at the site of the *TGF-β1* single-nucleotide polymorphism (SNP) (−509 C/T) was significantly associated with its plasma concentration. Yamada et al. described that *TGF-β1* SNPs + 29 T/C and −509 C/T alone [19] or in combination with −509 C/T and +869 T/C [20] combined with genetic susceptibility decreases the lumbar spine BMD and increases the prevalence of osteoporotic syndrome in postmenopausal Japanese women.

IL-10 is an anti-inflammatory cytokine that is secreted by activated immune cells. It limits the secretion of proinflammatory cytokines, such as TNF-α, IL-1, IL-6, IL-12, IL-2 and IFN-γ; modulates the differentiation and proliferation of immune cells, including macrophages, T cells and B cells; and attenuates monocyte recruitment or activation [21,22].

IL-10 also has potent inhibitory effects on osteoclastogenesis and acceleratory effects on osteoblastic differentiation [23]. In *IL-10* knockout mice, the hallmarks of osteoporosis can be observed, including an increased level of RANKL and osteoprotegerin, promoted bone resorption, reduced bone formation and altered trabecular structure [24,25]. Studies have shown that the *IL-10* gene polymorphisms may affect its mRNA and protein productions and are relevant in osteoporotic development. The genotype of the *IL-10* SNP (−627 C/C) correlated with a lower spinal BMD in postmenopausal osteoporotic patients [26].

In this study, we aimed to elucidate the correlation of risk factors, including age, height and genetic factors, for postmenopausal osteoporosis in Taiwanese. This study focuses on the interaction between four polymorphisms in both the *TGF-β1* and *IL-10* genes and the incidence of low BMD in postmenopausal osteoporotic women.

## 2. Materials and Methods

### 2.1. Study Participants

Osteoporotic patients were recruited after providing written informed agreement. The clinical trial protocol was approved by the Institutional Review Board of the Tri-Service General Hospital Medical Centre (TSGH-IRB #TC098-06). All subjects met the inclusion criteria based on BMD as an indicator of osteoporosis (T-score ≤ −2.5 standard deviations (SD)) or on a diagnosis of osteopenia with fracture (T-score < −1.0 and >−2.5 SD) using dual-energy X-ray absorptiometry (DXA) detection. Exclusion criteria for patients (in whom menses had stopped for more than 12 months) were as follows: (1) menolipsis at age < 40 years, or bilateral ovariectomies; (2) any medical history of illness, including primary hyperparathyroidism, overactive thyroid, diabetes, hepatic cirrhosis or renal failure; (3) drug treatment that affected bone metabolism, including bisphosphonates, calcitonin, glucocorticoids, thyroxin, or antiepileptics; or (4) hormone replacement therapy within the past 4 months [27,28]. After the study, osteoporotic patients were assessed for proper guideline-following osteoporotic treatment. Subjects without fracture (T-score > −1.0 SD) were representative control subjects. Written informed consent was obtained from each subject who agreed to have their deidentified medical information published. Each subject’s detailed medical history was considered and used for data analysis.

### 2.2. DNA Extraction and PCR

Venous blood samples collected from each subject and control individual were drawn and put into vacutainer tubes containing EDTA anticoagulant. To extract leukocyte DNA, an automated instrument with the MagNA Pure LC System (Roche Applied Science, Penzberg, Germany) was used. Genomic DNA (100 ng) was used for genotyping by polymerase chain reaction—restriction fragment length polymorphism (PCR-RFLP). The following reaction buffers were used: 1 × reaction buffer (1.5 mM MgCl_2_), 0.25 U DNA polymerase (JMR Holdings, Warwick, RI, USA), 0.4 μM each of forward primer and reverse primer (see the detailed sequences in Table 1) and 0.2 μM dNTP (Promega, Fitchburg, WI, USA). The Gene Amp@ PCR System 9700 (PE Applied Biosystem, Foster, CA, USA) was used with the following PCR cycles: first round predenaturation at 94 °C for 1 min, then by 35 cycles at 94 °C for 30 s, 62 °C for 30 s and 72 °C for 45 s followed by a final extension at 72 °C for 5 min and cooling to 4 °C.

### 2.3. PCR-RFLP Analysis

Four restriction enzymes (*Dde* I, *Bsr*B I, *Pvu* II and *Aci* I, New England BioLab, Beverly, MA, USA) were used for incubation with PCR products of the TGF-β1 SNPs (−509, +29, +869) and IL-10 SNP (+1927) at 37 °C overnight. Adequate numbers of informative bands were analyzed on a 3% high-resolution agarose gel (Thermo Fisher Scientific Inc., Waltham, MA, USA) containing ethidium bromide. The resulting images were photographed for genotyping (Table 1).

### 2.4. Assessments of BMD

A DXA bone densitometer (QDR 4500 W, Hologic, Bedford, MA, USA) was used in this study to assess the BMD values of the lumbar vertebra and bilateral hip joint of the whole body. Osteoporosis is defined as a disease in which the BMD (T-score) is lower than −2.5 SD below the average of a young healthy population, according to the WHO criteria [29].

### 2.5. Statistical Analysis

R2.15.1 software (R Foundation for Statistical Computing, Vienna, Austria) was used for statistical analysis. A two-tailed *p* value less than 0.05 was considered statistically significant. The distributional properties of the continuous variables were represented as the means ± SD and categorical variables were expressed relating to frequency and percentage. In the univariate analysis, the continuous variables of normal distributions were compared between the healthy and osteoporotic subjects with a two-sample *t* test. Continuous variables that did not fit normal distributions were examined by the Mann–Whitney *U* test (Wilcoxon rank-sum test). The chi-squared test or Fisher’s exact test was used to compare categorical variables between groups. In the multivariate analysis, the test was conducted by fitting linear regression models to the effects of risk factors on four continuous outcome variables (BMD T-score for the total hip, lumbar spine and femoral neck and height).

The regression analysis procedures followed the recommended approach [30,31]. Briefly, to find a parsimonious regression model that fit our data well for effect estimation and outcome prediction, well-known model-fitting techniques, including variable selection, goodness-of-fit (GOF) assessment and regression diagnostics and remedies, were used. In this study, the linear regression model was used and conducted with a stepwise variable selection procedure, and all the selected univariate significant and non-significant relevant covariates and some of their interactions are listed in Table 2. To acquire the best candidate final linear regression model, we used a conservative measurement (the significance levels for entry (SLE) and for stay (SLS) were set to ≥0.15) and then used substantive knowledge to reduce the non-significant covariates (*p* value > 0.05) one at a time until all regression coefficients were significantly different from 0. In addition, the coefficient of determination (*R*^2^, 0 ≤ *R*^2^ ≤ 1) was also examined to evaluate the GOF of the fitted regression model. A value of *R*^2^ near 1 indicates that most of the response variability is explained by the covariates used in the multiple linear regression model [27,32].

## 3. Results

### 3.1. Analysis of SNPs within the TGF-β1 and IL-10 Genes

**Figure 1:**
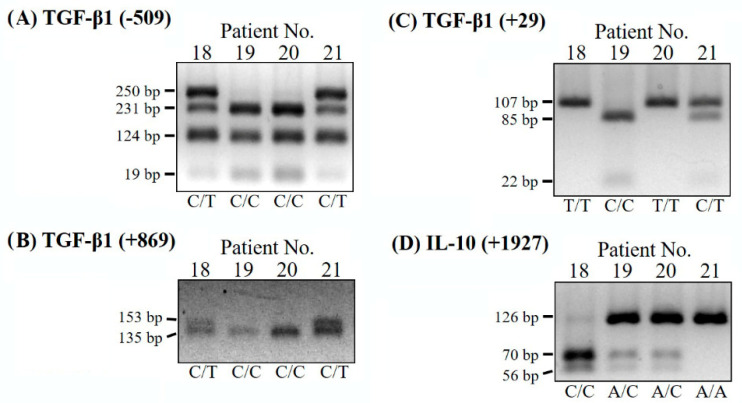
Representative electrophoresis gel images of the PCR-RFLP analysis of Taiwanese osteoporotic patients. The different single-nucleotide polymorphisms (SNPs) of (**A**) *TGF-β1* (−509 C/T), (**B**) *TGF-β1* (+869 T/C), (**C**) *TGF-β1* (+29 T/C) and (**D**) *IL-10* (+1927 A/C) are shown for patients No. 18 to No. 21.

Three SNPs of the *TGF-β1* gene, including *TGF-β1* (−509) at the promoter region, *TGF-β1* (+29) and *TGF-β1* (+869) at the exon 1 region and one SNP of *IL-10* (+1927) at the exon 2 region, were analyzed in this study. Blood DNA obtained from patients with osteoporosis/osteopenia with fractures and from healthy subjects were individually amplified by four pairs of SNP PCR primers, as shown in Table 1. PCR-based RFLP analyses were performed with the digestion of different restriction enzymes: *Dde* I for *TGF-β1* SNP (−509 C/T), *Bsr*B I for *TGF-β1* SNP (+29 C/T), *Pvu* II for *TGF-β1* SNP (+869 C/T) and *Aci* I for *IL-10* SNP (+1927 A/C). The enzyme-digested fragment-length polymorphisms were predicted as shown in Table 1 and the representative results are shown in Figure 1. Interestingly, a strong linkage of the three *TGF-β1* SNPs (−509 T/C, +29 T/C, and +869 T/C) in each patient was found in the *TGF-β1* SNPs: −509 C/C linked with the SNP (+29 T/T) and SNP (+869 C/C) types, −509 C/T linked with the SNP (+29 C/T) and SNP (+869 C/T) types, and −509 T/T linked with the SNP (+29 C/C) and SNP (+869 T/T) and types, respectively (Supplementary Materials Table S1). We also checked the linkage disequilibrium (LD) between the *TGF-β1* SNPs in the identified loci using the LDpair tool (https://ldlink.nci.nih.gov/?tab=ldpair, accessed on 29 November 2020). The −509 T/C and +29 T/C SNPs showed a strong linkage disequilibrium with a D’ > 0.99. These SNPs also showed strong linkage disequilibrium in the studies of Langdahl et al. [12] and Tzakas et al. [33]. The LD effects demonstrated that these *TGF-β1* SNPs may therefore be caused by one of them and influence each other. Thus, we evaluated the patients in terms of the SNPs, *TGF-β1* SNP (−509) and *IL-10* SNP (+1927), which can also simultaneously represent the effects of the *TGF-β1* SNPs (+29 and +869) on the risk of osteoporosis.

### 3.2. Characteristics of the Study Population

A total of 222 subjects were recruited in the beginning, but five subjects in the osteoporosis group were excluded for their use of glucocorticoid or thyroxin drugs. Thus, finally, 88 osteoporotic patients and 129 healthy control subjects were enrolled in this study. The means for gender, anthropometric parameters (age, weight, height and corresponding body mass index), BMD T-scores, vegetarian status, habitual dietary calcium (Ca) and *TGF-β1* SNP (−509) and *IL-10* SNP (+1927) for patients with osteoporosis (*n* = 88) and control subjects (*n* = 129) are shown in Table 2. No significant difference was observed in BMI, gender or vegetarian status (*p* > 0.05) between the healthy controls and the patients with osteoporosis or osteopenia with fractures. The genotype frequency of the *TGF-β1* SNP (−509) or *IL-10* SNP (+1927) showed no significant difference between the healthy control subjects and the patients with osteoporosis (*p* = 0.838 vs. *p* = 0.439, respectively). However, the bone fracture patients were significantly shorter (*p* < 0.001) and weighed less (*p* = 0.008) than the healthy control subjects in univariate analysis (Table 2). The mean values of Ca intake and T-scores for the lumbar spine, total hip, and femoral neck were all lower in osteoporotic patients than in healthy subjects (*p* < 0.001). In addition, the DXA BMD values for spine, femoral neck, and total hip in patients with osteoporosis or osteopenia with fractures were significantly lower than that in the control subjects (*p* < 0.001), as shown in Supplementary Materials Table S2. The BMD T-scores, habitual dietary calcium, age, weight and height in the patients we studied showed a significant difference between healthy controls.

### 3.3. Multivariate Analysis of SNPs and BMD Correlation Using a Linear Regression Model

To identify the predictors of the four continuous outcome variables, a multivariate analysis of the BMD T-scores was performed by fitting four multiple linear regression (MLR) models for lumbar spine (Y1), femoral neck (Y2), total hip (Y3) and height (Y4), as shown in Table 3, Table 4, Table 5 and Table 6. Body height appeared as an independent positive predictor, which was shown in the MLR models of Y1, Y2 and Y3; therefore, it has been added as an outcome variable in the regression models. Otherwise, body height could also act as an intermediate variable in the pathways linking sources between some SNPs and T-scores (Y1, Y2 and Y3).

### 3.4. Association of Combined Polymorphisms with Lumbar Spine BMD in Osteoporotic Patients

The multiple-variable linear regression analysis results of the possible factors associated with the spinal BMD T-score (Y1) in those with osteoporosis or osteopenia with fractures and *TGF-β1* (−509 T/C) and *IL-10* (+1927 A/C) are shown in Table 3. After adjustment for the covariates in the MLR model, subjects who were 1 cm taller had a positive corresponding increase of 0.0577 in their spinal BMD T-score (Y1; *p* < 0.0001). Daily calcium (Ca) intake greater than 800 mg/day increased the spinal T-score by 0.3764 (*p* < 0.05). Younger age (33–55 years old) was also found to be a beneficial factor for osteoporosis, associated with an increase in spinal T-scores of 0.6541 (*p* < 0.001). After adjusting for the effects of the other covariates, the estimated mean value of the spinal BMD T-score, Y1, in the subjects with an age between 33 and 55 years would be 0.6541 higher than those younger than 33 years or older than 55 years. After adjustment for the covariates of height, age and Ca intake, the patients with both polymorphisms of the *TGF-β1* SNP (−509 T/T) and *IL-10* SNP (+1927 A/A + A/C) genotypes and the female patients with both polymorphisms of the *TGF-β1* SNP (−509 C/C) and *IL-10* SNP (+1927 C/C) genotypes had spinal T-scores (Y1) of 0.4242 and 1.0417, respectively; however, these two interactions showed no significant difference (*p* = 0.0759 and 0.1356, respectively). A higher level of spinal BMD T-scores could be found in younger age (33–55 years old), taller people and people with Ca intake > 800 mg/day, but there was no significant relation connected to the *TGF-β1* SNP (−509 C/C) and *IL-10* SNP (+1927 C/C) genotypes.

### 3.5. Association of Combined SNPs with Femoral Neck BMD in Osteoporotic Patients

The multiple-variable linear regression analysis results of the correlations between the BMD T-score of femoral neck (Y2) and the polymorphisms of *TGF-β1* (−509 T/C) and *IL-10* (+1927 A/C) in patients are shown in Table 4. After adjustment for the covariates in the MLR model, 33 < age ≤ 68 (0.4654, *p* < 0.05), body height (0.0551, *p* < 0.0001), and Ca intake > 855 mg/day (0.3726, *p* < 0.05) had beneficial effects on the mean value of the femoral neck T-score (Y2). The femoral neck T-scores in female patients and in middle-aged patients (≥55 years) were 0.5283 lower than those in the male patients of the same age (*p* < 0.01). Considering the interaction of gender and height, the femoral neck T-scores in female patients were 0.0049 higher than those in the male patients of the same height (*p* < 0.05).

The femoral neck T-score of female subjects with both polymorphisms of the *TGF-β1* SNP (−509 C/C) and *IL-10* SNP (+1927 C/C) genotypes was 1.1666 higher than those in any other group (*p* < 0.05), which means these genotypes had a greater tendency to prevent osteoporosis. However, there was only a borderline significant increase in the female subjects with both polymorphisms of the *TGF-β1* SNP (−509 T/T) and *IL-10* SNP (+1927 C/C + A/C) genotypes and the results showed a 0.4220 higher femoral neck T-score in these patients than in any other group (*p* = 0.0559). A higher level of femoral neck BMD T-scores could be found in the age range of 33–68 years old, in taller people, females, people with Ca intake > 855 mg/day, and the *TGF-β1* SNP (−509 C/C) and *IL-10* SNP (+1927 C/C) genotypes, but there was no significant relation connected to the *TGF-β1* SNP (−509 T/T) and *IL-10* SNP (+1927 C/C + A/C) genotypes.

### 3.6. Association of Combined SNPs with Total Hip BMD in Osteoporotic Patients

The multiple-variable linear regression analysis of the correlations between the total hip BMD T-score (Y3) and the polymorphisms *TGF-β1* SNP (−509 T/C) and *IL-10* SNP (+1927 A/C) in patients are shown in Table 5. After adjustment for the covariates in the MLR model, body height (0.0331, *p* < 0.05) and Ca intake > 875 mg daily (0.4561, *p* < 0.01) had beneficial effects on the mean value of the total hip BMD T-score (Y3). Considering the interaction of gender and age, the total hip T-score in female subjects was 0.0560 lower than those of male subjects of the same age (*p* < 0.0001). Considering the interaction of gender and height, the total hip T-scores in female patients were 0.0229 higher than that of male patients of the same height (*p* < 0.0001). The total hip T-score of subjects with both polymorphisms of the *TGF-β1* SNP (−509 T/T) and *IL-10* SNP (+1927 A/A) genotypes was 1.4692 lower than that in any other group (*p* < 0.01). A higher level of total hip BMD T-scores could be found in taller people and people with Ca intake > 875 mg/day. Females who had the same height as males showed a higher T-score, but a lower T-score was found in females compared to males who were of the same age. A lower level of total hip BMD T-scores could be found for the *TGF-β1* SNP (−509 T/T) and *IL-10* SNP (+1927 A/A) genotypes.

### 3.7. Association of Combined SNPs with Body Height

The multiple-variable linear regression analysis of the possible correlations between body height (Y4) and the polymorphisms *TGF-β1* SNP (−509 T/C) and *IL-10* SNP (+1927 A/C) in patients with osteoporosis or osteopenia with fractures are shown in Table 6. After adjustment for the covariates in the MLR model, females who had these SNP genotypes were 11.404 cm shorter than those in any other group (*p* < 0.0001) and patients who had these SNP genotypes had a one-year age difference associated with a 0.3503 cm shorter body height (*p* < 0.0001).

Patients with the polymorphism *TGF-β1* SNP (−509 C/T) genotype were 1.8464 cm taller than those in any other group (*p* < 0.05). Females with both polymorphisms of the *TGF-β1* SNP (−509 C/C + C/T) and *IL-10* SNP (+1927 C/C) genotypes were 4.3845 cm shorter than the female subjects in any other group (*p* < 0.01). However, males who had both polymorphisms of the *TGF-β1* (−509 T/T) and *IL-10* (+1927 A/C) genotypes were 7.8140 cm taller than those in any other group (*p* < 0.01).

## 4. Discussion

Osteoporosis is a progressive disease with a multifactorial etiology that also represents an increasingly serious health problem around the world. Although the variation in BMD, a major index determining bone strength, is determined by environmental, behavioral, hormonal and nutritional factors, the most important component of the variation in BMD may be genetically controlled [1,34]. Although many pathogenic genes/SNPs have been identified to be associated with the BMD T-score or osteoporosis-related phenotypes, their results are still not consistent and lack robustness [35,36].

TGF-β1 is an abundant protein in the bone matrix and is stored in an inactive precursor form until the initiation of bone resorption, in which TGF-β1 will be released and activated by the acidic environment of osteoclasts in resorption lacunae [37]. Mounting evidence has shown that estrogen may enhance osteoclast apoptosis, increasing both the active and latent TGF-β1 levels and then participating in the regulation of bone formation [14,38]. Considering that sex hormones may affect bone remodeling and BMD data, we analyzed both males and females in this study. Indeed, the femoral neck T-scores in female patients and in middle-aged (≥55 years) patients showed a lower BMD than those of male patients of the same age. Moreover, the femoral neck T-score of female subjects with both polymorphisms of the *TGF-β1* (−509 C/C) and *IL-10* (+1927 C/C) genotypes showed the greatest tendency to prevent osteoporosis compared with those of all other groups (Table 4).

There are three SNPs in the *TGF-β1* gene, namely, −509 C/T, +29 T/C and +869 T/C, that have been associated with susceptibility to osteoporosis. No association between the *TGF-β1* polymorphisms (−509 T/C, rs1800469; +29 T/C, rs1800470 and +869 T/C, rs1982073) and *IL-10* polymorphism (+1927 A/C, rs3021094) and osteoporosis/BMD can be found in the NHGRI-EBI Catalog of published genome-wide association studies (GWAS; https://www.ebi.ac.uk/gwas/, accessed on 27 November 2020). Langdahl et al. [38] used the GENOMOS study for a large-scale analysis of the association between these three *TGF-β1* polymorphisms and osteoporosis, which showed that these polymorphisms were associated with BMD reduction or fracture risk increment in some populations. Accumulating evidence has shown that the SNP + 29 T→C transition in the signal sequence of the anti-inflammatory *TGF-β1* gene is significantly associated with higher BMD at the spine and hip in Japanese and German women [39,40], but not in white women in the United States [41]. Although previous studies showed some contradictory results for this polymorphism on BMD, the genotype and haplotype frequencies in a Florence cross-sectional study showed that the T-allele of the −509 T/C polymorphism and C-allele of the +29 T/C polymorphism were highly associated with a reduced BMD; otherwise, the CT + CC genotype of the *TGF-β1* (+869 T/C) polymorphism was associated with increased risk of osteoporotic fracture at the lumbar spine and femoral neck [38,42]. Sun et al. [13] performed a systematic online search that revealed *TGF-β1* (+869 T/C) and (+29 T/C) polymorphisms increased the susceptibility to osteoporosis in an Asian population. The combination of both the *TGF-β1* (−509 C/T) and (+869 T/C) polymorphisms was associated with the risk of osteoporosis [19,20], IgA nephropathy (IgAN) [43] and urinary tract infection and vesicoureteral reflux (VUR) [44]. In this study, we found a strong association of the three *TGF-β1* SNPs (−509 T/C, +29 T/C and +869 T/C) in each patient (Supplementary Table S1). Thus, we evaluated the patients in terms of the SNPs *TGF-β1* (−509) and *IL-10* (+1927), which can also represent the effects of *TGF-β1* SNPs (+869 and +9) on the risk of osteoporosis at the same time.

The polymorphic genotype of the anti-inflammatory *IL-10* gene could affect protein expression and activity, therefore shifting the balance between bone formation and resorption and increasing the subsequent risk of osteoporosis. Animal studies have shown that a lack of *IL-10* expression decreases femoral BMD, increases bone fragility and inhibits bone resorption, eventually leading to osteoporosis [24]. Park et al. [45] reported that the *IL-10* SNP (−592 C/C) and/or *IL-10* ht2 (A-C-C-T) genotypes generally had relatively lower spinal BMD in Korean postmenopausal women. In addition, mounting studies have shown that the presence of the C allele at position—597 decreased the frequency of the G allele at position—1082, or a polymorphism of the C/C genotype at position—627 in the *IL-10* promoter may constitute a risk for the development of osteoporosis [8,26,46]. The SNP at the splice region of *IL-10* (+1927) showed that the A/A genotype was related to a higher risk for hepatocellular carcinoma [47,48]. However, the biological significance of the *IL-10* (+1927) polymorphism in osteoporosis has not been revealed until now.

Bone loss caused by the development of osteoporosis may be gradual and painless with no significant clinical symptoms. In this study, we found that bone loss also triggered a higher ratio of different types of fractures in osteoporosis or osteopenia patients (Supplementary Materials Table S3). Osteoporosis disease may progress silently until a fracture occurs or kyphosis presents. Osteoporotic patients can lose up to 4 inches in body height and also may suffer severe chronic pain as a result of the bending or collapse of the spine [49]. However, the correlations between body height and *TGF-β1* or *IL-10* gene polymorphisms in osteoporotic development have not yet been revealed. In this study, we identified body height as another predictor in the causal pathway between SNPs and spinal, femoral neck, and total hip T-scores. Furthermore, the *TGF-β1* SNP (−509 C/T) genotype in all subjects and the *TGF-β1* SNP (−509 T/T) and *IL-10* SNP (+1927 A/C) genotypes in males showed positive associations with body height.

## 5. Conclusions

This study is the first report to evaluate the risks of osteoporosis and the associations between the anti-inflammatory cytokine-specific SNPs—*TGF-β1* SNP (−509 T/T) and *IL-10* SNP (+1927 A/A)—and adjusted age, habitual Ca intake, gender, BMI and height in Taiwanese subjects; it showed a significantly lower total hip BMD when compared with other SNP combinations. Furthermore, we also found that the combination of both polymorphisms of the *TGF-β1* SNP (−509 C/C) and *IL-10* SNP (+1927 C/C) genotypes showed a higher femoral neck BMD T-score in female subjects than in any other group, which means these genotypes had a potential to act as a predictive factor for postmenopausal osteoporosis in Taiwanese women. Therefore, early genetic testing of these predictive factors for postmenopausal osteoporosis in the Taiwanese female population can help to prescribe to patients with these factors the appropriate nutritional supplements or clinical treatments, eventually slowing down the bone mass loss caused by age and inheritance and preventing osteoporosis-related diseases. However, well-designed studies with larger sample sizes are required to further explicate these associations.

## Figures and Tables

**Table 1:** Description of the four SNPs within the *TGF-β1* and *IL-10* genes and the prediction of genotypes by PCR-RFLP fragments.

SNPs	Oligonucleotide PRIMERS (F: Forward Primer; R: Reverse Primer)	Restriction Enzyme (PCR Size)	PCR-RFLP Fragments (bp)	Genotype
TGF-β1 SNP (−509 C/T)	F: 5′-CCAGCTAAGGCATGGCACCG-3′ R: 5′-GCGGTGTGGGTCACCAGAGA-3′	*Dde* I (380 bp)	231, 124, 19, 6 250, 231,124, 19, 6 250, 124, 6	C/C C/T T/T
TGF-β1 SNP (+29 C/T)	F: 5′-ACCACACCAGCCCTGTTCGCGC-3′ R: 5′-AGCCACAGCAGCGGTAGCAGGA-3′	*BsrB* I (107 bp)	107 107, 85, 22 85, 22	T/T C/T C/C
TGF-β1 SNP (+869 C/T)	F: 5′-TCCGGGCTGCGGCTGCAGC-3′ R: 5′-CAGGATCTGGCCGCGGATGG-3′	*Pvu* II (153 bp)	153 153, 135, 18 135, 18	T/T C/T C/C
IL-10 SNP (+1927 A/C)	F: 5′-GCTTCTGCTTTCCCTTCAAAAT-3′ R: 5′-AAATCAAAAGGGGAGTTTTAAA-3′	*Aci* I (126 bp)	126 126, 70, 56 70, 56	A/A A/C C/C

**Table 2:** Clinical characteristics of patients with osteoporosis or osteopenia with fractures and healthy controls.

Parameter ^1^	Patients with Osteoporosis/Osteopenia with Fractures (*n* = 88)	Controls (*n* = 129)	*p*-Value ^2^
Sample Size (*n*)	88/217 (40.6%)	129/217 (59.4%)	
Gender			0.534
Male	21/57 (36.8%)	36/57 (63.2%)	
Female	67/160 (41.9%)	93/160 (58.1%)	
Age (years old)	65.69 ± 13.12	57.71 ± 10.44	<0.001
Height (cm)	154.72 ± 9.27	160.69 ± 7.25	<0.001
Weight (kg)	58.53 ± 10.41	62.62 ± 9.81	0.008
BMI (kg/m^2^)	24.48 ± 4.18	24.26 ± 3.50	0.786
Ca Intake (mg/day)	697.20 ± 234.11	822.16 ± 271.25	0.001
Vegetarian			0.064
Yes	6/8 (75.0%)	2/8 (25.0%)	
No	82/209 (39.2%)	127/209 (60.8%)	
*TGF-β1* SNP (−509)			0.838
C/C	13/35 (37.1%)	22/35 (62.9%)	
C/T	52/121 (43.0%)	69/121 (57.0%)	
T/T	23/61 (37.7%)	38/61(62.3%)	
*IL-10* SNP (+1927)			0.439
A/A	22/64 (34.4%)	42/64 (65.6%)	
A/C	43/98 (43.9%)	55/98 (56.1%)	
C/C	23/55 (41.8%)	32/55 (58.2%)	
Lumbar Spine T-Score	−2.01 ± 1.14	−0.29 ± 1.10	<0.001
Femoral Neck T-Score	−2.19 ± 0.85	−0.61 ± 0.91	<0.001
Total Hip T-Score	−1.66 ± 1.03	0.03 ± 0.90	<0.001

^1^ The statistics are presented as the means ± SD for continuous variables and frequency (%) for categorical variables. ^2^ The *p* values were calculated using the Fisher’s exact test for categorical variables and the Wilcoxon rank-sum test for continuous variables.

**Table 3:** Multivariate analyses of the predictors of spinal bone mineral density (BMD) T-scores using MLR models.

Covariate	Estimate	Standard Error	*t*-Value	*p*-Value
Y1 = Spinal BMD T-Score ^1^				
Intercept	−10.5181	1.8483	−5.691	<0001
Height (cm)	0.0577	0.0116	4.970	<0001
33 < Age ≤ 55 (years old)	0.6541	0.1928	3.393	0.0009
Ca Intake > 800 (mg/day)	0.3764	0.1869	2.014	0.0458
*TGF-β1* SNP (−509 T/T) × *IL-10* SNP (+1927 A/A + A/C)	0.4242	0.2374	1.787	0.0759
Female × *TGF-β1* SNP (−509 C/C) × *IL-10* SNP (+1927 C/C)	1.0417	0.6944	1.500	0.1356

^1^ Number of observations = 158; the coefficient of determination (*R*^2^) was 0.2628, indicating that the correlation between the observed response value and the predicted value of the response variable was approximately 0.51.

**Table 4:** Multivariate analyses of the predictors of femoral neck BMD T-scores using MLR models.

Covariate	Estimate	Standard Error	*t*-Value	*p*-Value
Y2 = Femoral Neck BMD T-Score ^1^				
Intercept	−10.7755	1.9377	−5.561	<0.0001
30 < Age ≤ 68 (years old)	0.4654	0.2055	2.265	0.0248
Height (cm)	0.0551	0.0119	4.642	<0.0001
Ca Intake ≥ 855 (mg/day)	0.3726	0.1499	2.485	0.0140
Female × Age ≥ 55 (years old)	−0.5283	0.1929	−2.739	0.0068
Height (cm) × Female	0.0049	0.0016	3.093	0.0023
Female × *TGF-β1* SNP (−509 C/C) × *IL-10* SNP (+1927 C/C)	1.1666	0.5850	1.994	0.0478
Female × *TGF-β1* SNP (−509 T/T) × *IL-10* SNP (+1927 C/C + A/C)	0.4220	0.2191	1.926	0.0559

^1^ Number of observation subjects = 171; the coefficient of determination (*R*^2^) was 0.3629, indicating that the correlation between the observed response value and the predicted value of the response variable was approximately 0.60.

**Table 5:** Multivariate analyses of the predictors of total hip BMD T-scores using MLR models.

Covariate	Estimate	Standard Error	*t*-Value	*p*-Value
Y3 = Total Hip BMD T-Score ^1^				
Intercept	−6.1780	2.2805	−2.709	0.0075
Height (cm)	0.0331	0.0137	2.422	0.0165
Ca Intake > 875 (mg)	0.4561	0.1532	2.976	0.0034
Height (cm) × Female	0.0229	0.0034	6.658	<0.0001
Female × Age (years)	−0.0560	0.0102	−5.509	<0.0001
*TGF-β1* SNP (−509 T/T) × *IL-10* SNP (+1927 A/A)	−1.4692	0.4704	−3.123	0.0021

^1^ Number of observation subjects = 171; the coefficient of determination (*R*^2^) was 0.3639, indicating that the correlation between the observed response value and the predicted value of the response variable was approximately 0.60.

**Table 6:** Multivariate analyses of the predictors of height using MLR models.

Covariate	Estimate	Standard Error	*t*-Value	*p*-Value
Y4 = Height (cm) ^1^				
Intercept	187.3222	2.5461	73.573	<0.0001
Female × *TGF-β1* SNP (−509 T/C) × *IL-10* SNP (+1927 A/C)	−11.4040	1.0245	−11.132	<0.0001
Age × *TGF-β1* SNP (−509 T/C) × *IL-10* SNP (+1927 A/C)	−0.3503	0.0381	−9.208	<0.0001
*TGF-β1* SNP (−509 C/T)	1.8464	0.8926	2.069	0.0402
Female × *TGF-β1* SNP (−509 C/C + C/T) × *IL-10* SNP (+1927 C/C)	−4.3845	1.4081	−3.114	0.0022
Male × *TGF-β1* SNP (−509 T/T) × IL-10 SNP (+1927 A/C)	7.8140	2.7417	2.850	0.0049

^1^ Number of observation subjects = 171; the coefficient of determination (*R*^2^) was 0.5859, indicating that the correlation between the observed response value and the predicted value of the response variable was approximately 0.77.

## Data Availability

The data presented in this study are available on request from the corresponding author.

## Supplementary Materials

The following are available online at https://www.mdpi.com/article/10.3390/genes12060930/s1, Table S1: PCR-RFLP results obtained from each patient; Table S2. Bone mineral density (BMD) of patients with osteoporosis and control subjects determined by a dual-energy X-ray absorptiometry (DXA); Table S3. The frequency and location of fractures in the subjects of this cohort study.
