# Supplementary material for: Association of TGF-β1 and IL-10 Gene Polymorphisms with Osteoporosis in a Study of Taiwanese Osteoporotic Patients
Source: Genes (Basel). 2021 Jun 18;12(6):930. doi: 10.3390/genes12060930 (PMC8233820; doi:10.3390/genes12060930)
Supplement: Supplementary file 1 [file genes-12-00930-s001.zip › genes-1242056-supplementary.pdf]

Supplementary Table S1. PCR-RFLP results obtained from each patient

Patient No.	TGF-β1 SNP		
	-509	+869	+29
1	C/C	C/C	T/T
2	C/T	C/T	C/T
3	T/T	T/T	C/C
4	C/C	C/C	T/T
5	C/T	C/T	C/T
6	C/T	C/T	C/T
7	C/C	C/C	T/T
8	C/T	C/T	C/T
9	C/T	C/T	C/T
10	C/T	C/T	C/T
11	C/T	C/T	C/T
13	T/T	T/T	C/C
14	C/T	C/T	C/T
15	T/T	T/T	C/C/
16	C/T	C/T	C/T
17	T/T	T/T	C/C
18	C/T	C/T	C/T
19	C/C	C/C	T/T
20	C/C	C/C	T/T
21	C/T	C/T	C/T
22	T/T	T/T	C/C
23	T/T	T/T	C/C
24	C/T	C/T	C/T
25	C/C	C/C	T/T
26	C/T	C/T	C/T
27	C/T	C/T	C/T
28	C/T	C/T	C/T
29	C/T	C/T	C/T
30	C/T	C/T	C/T
31	T/T	T/T	C/C
32	T/T	T/T	C/C
33	C/T	C/T	T/T
34	T/T	T/T	C/C
35	T/T	T/T	C/C
36	C/C	C/C	T/T
37	T/T	T/T	C/C
38	C/C	C/C	T/T
39	C/T	C/T	C/T
40	C/T	C/T	C/T
41	C/T	C/T	C/T
42	C/T	C/T	C/T

Supplementary Table 2. Bone mineral density (BMD) of patients with osteoporosis and control subjects determined by a dual-energy X-ray absorptiometry (DXA)

Parameter	Patients with Osteoporosis/Osteopenia with Fractures	Controls	*p*-Value
DXA BMD (g/cm^3)			
Spine	0.84 ± 0.15	1.05 ± 0.11	< 0.001
Hip Neck	0.60 ± 0.11	0.79 ± 0.10	< 0.001
Total Hip	0.71 ± 0.14	0.92 ± 0.10	< 0.001

Supplementary Table 3. The frequency and location of fractures in the subjects of this cohort study

Parameter	Patients with Osteoporosis/Osteopenia with Fractures	Controls
Location of fractures		
Spine	32/88 (36.36%)	0/129
Hip	18/88 (20.45%)	0/129
Wrist shoulder	29/88 (32.95%)	0/129
Thigh leg	12/88 (13.64%)	0/129
